# Supplementary material for: Behaviourally modulated hippocampal theta oscillations in the ferret persist during both locomotion and immobility
Source: Nat Commun. 2022 Oct 7;13:5905. doi: 10.1038/s41467-022-33507-2 (PMC9547075; doi:10.1038/s41467-022-33507-2)
Supplement: Supplementary file 3 — Reporting Summary [file 41467_2022_33507_MOESM3_ESM.pdf]

nature portfolio

Corresponding author(s): Soraya Dunn
Jennifer Bizley
Daniel Bendor

Last updated by author(s): Sep 6, 2022

Reporting Summary

Nature Portfolio wishes to improve the reproducibility of the work that we publish. This form provides structure for consistency and transparency in reporting. For further information on Nature Portfolio policies, see our Editorial Policies and the Editorial Policy Checklist.

Statistics

For all statistical analyses, confirm that the following items are present in the figure legend, table legend, main text, or Methods section.

n/a	Confirmed	
☐	☒	The exact sample size (n) for each experimental group/condition, given as a discrete number and unit of measurement
☐	☒	A statement on whether measurements were taken from distinct samples or whether the same sample was measured repeatedly
☐	☒	The statistical test(s) used AND whether they are one- or two-sided<br>*Only common tests should be described solely by name; describe more complex techniques in the Methods section.*
☐	☒	A description of all covariates tested
☐	☒	A description of any assumptions or corrections, such as tests of normality and adjustment for multiple comparisons
☐	☒	A full description of the statistical parameters including central tendency (e.g. means) or other basic estimates (e.g. regression coefficient) AND variation (e.g. standard deviation) or associated estimates of uncertainty (e.g. confidence intervals)
☐	☒	For null hypothesis testing, the test statistic (e.g. F, t, r) with confidence intervals, effect sizes, degrees of freedom and P value noted<br>*Give P values as exact values whenever suitable.*
☒	☐	For Bayesian analysis, information on the choice of priors and Markov chain Monte Carlo settings
☐	☒	For hierarchical and complex designs, identification of the appropriate level for tests and full reporting of outcomes
☒	☐	Estimates of effect sizes (e.g. Cohen's d, Pearson's r), indicating how they were calculated

Our web collection on statistics for biologists contains articles on many of the points above.

Software and code

Policy information about availability of computer code

Data collection	Data were collected using Neuralynx Cheetah 5.7.4 acquisition software, TDT System 3 (OpenEx v90), Multi Channel DataManager v1.5.0.0, and custom written MATLAB code (MATLAB 2014b).
Data analysis	Data analysis was performed using custom scripts in MATLAB versions 2014a-2020a (available on GitHub https://github.com/slsdunn/theta-paper-code) and R, IMOD 3dmod 4.9.8., CircStat MATLAB toolbox (Berens 2009), LME4 R package v1.1-30, emmeans R package v 1.8.0

For manuscripts utilizing custom algorithms or software that are central to the research but not yet described in published literature, software must be made available to editors and reviewers. We strongly encourage code deposition in a community repository (e.g. GitHub). See the Nature Portfolio guidelines for submitting code & software for further information.

Data

Policy information about availability of data

All manuscripts must include a data availability statement. This statement should provide the following information, where applicable:

- Accession codes, unique identifiers, or web links for publicly available datasets
- A description of any restrictions on data availability
- For clinical datasets or third party data, please ensure that the statement adheres to our policy

The processed electrophysiological and behavioural data has been deposited at Figshare (DOI 10.5522/04/21070128). Data presented in all figures are available at Figshare (DOI 10.5522/04/20809099).

Field-specific reporting

Please select the one below that is the best fit for your research. If you are not sure, read the appropriate sections before making your selection.

☒ Life sciences ☐ Behavioural & social sciences ☐ Ecological, evolutionary & environmental sciences

For a reference copy of the document with all sections, see nature.com/documents/nr-reporting-summary-flat.pdf

Life sciences study design

All studies must disclose on these points even when the disclosure is negative.

Sample size	Power calculations weren't performed as this is exploratory research; however numbers of animals chosen were sufficient to test repeatability (based on previous similar hippocampal work in rats eg n = 3 in https://doi.org/10.1016/j.neuron.2021.01.017; work with ferrets eg n=2 in https://www.nature.com/articles/nn1141; and other non-rodent hippocampal work eg macaques n=3 in https://doi.org/10.1016/j.neuron.2021.09.032).
Data exclusions	No animals were excluded from the study. Data from some experimental sessions were excluded based on equipment failures or noise levels. Noisy portions of neural data were excluded within sessions using an automated cleaning algorithm as described in the methods.
Replication	Repeated measurements were taken from each animal and results across sessions were consistent. The exact number for each condition is in Supplementary Table 1.
Randomization	Experimental groups were different species so randomisation was not applicable. The only group wise comparison was performed across species and it was not possible to assign individuals randomly to species.
Blinding	The investigators were blind to the precise anatomical location of probes and LFP power during testing. Behavioural testing was fully automated and randomised by custom written software (animals were monitored for safety). Automated analysis routines were performed blind to the recording site/probe/animal.

Reporting for specific materials, systems and methods

We require information from authors about some types of materials, experimental systems and methods used in many studies. Here, indicate whether each material, system or method listed is relevant to your study. If you are not sure if a list item applies to your research, read the appropriate section before selecting a response.

Materials & experimental systems

n/a	Involved in the study
☒	☐ Antibodies
☒	☐ Eukaryotic cell lines
☒	☐ Palaeontology and archaeology
☐	☒ Animals and other organisms
☒	☐ Human research participants
☒	☐ Clinical data
☒	☐ Dual use research of concern

Methods

n/a	Involved in the study
☒	☐ ChIP-seq
☒	☐ Flow cytometry
☒	☐ MRI-based neuroimaging

Animals and other organisms

Policy information about studies involving animals; ARRIVE guidelines recommended for reporting animal research

Laboratory animals	3 adult male Lister hooded rats (aged 2 - 10 months) and 4 adult female pigmented ferrets (aged 4-19 months) were used in this study.
Wild animals	This study did not involve wild animals.
Field-collected samples	The study did not involve samples collected from the field.
Ethics oversight	All experimental procedures performed were first approved by a local ethical review committee at University College London. Procedures were carried out under license from the UK Home Office in accordance with the Animals (Scientific Procedures) Act (1986) and PPLs 70/8636 and 70/8637 for rat procedures and PPL 70/7278 and 70/7267 for ferret procedures.

Note that full information on the approval of the study protocol must also be provided in the manuscript.
